# The incidence of malignant brain tumors is increased in patients with obstructive sleep apnea: A national health insurance survey

**DOI:** 10.1371/journal.pone.0241598

**Published:** 2020-11-12

**Authors:** Jae Hoon Cho, Young Chang Lim, Kyung-Do Han, Jae Yong Lee, Ji Ho Choi

**Affiliations:** 1 Department of Otorhinolaryngology-Head and Neck Surgery, College of Medicine, Konkuk University, Seoul, Republic of Korea; 2 Department of Statistics and Actuarial Science, Soongsil University, Seoul, Republic of Korea; 3 Department of Otorhinolaryngology-Head and Neck Surgery, Bucheon Hospital, Soonchunhyang University College of Medicine, Bucheon, Republic of Korea; Goethe University Hospital Frankfurt, GERMANY

## Abstract

The association between obstructive sleep apnea (OSA) and malignant brain tumors has yet to be fully investigated. Therefore, the purpose of this study was to elucidate the effect of OSA on brain tumor incidence based on the Korea National Health Insurance Service (KNHIS) dataset. The KNHIS data between 2007 and 2014 were analyzed, and the primary endpoint was newly diagnosed malignant brain tumor. A total of 198,574 subjects aged ≥ 20 years with newly diagnosed OSA were enrolled in the study, and 992,870 individuals were selected as a control group based on propensity score matching (PSM) by gender and age. The average follow-up duration was 4.8 ± 2.3 years. The hazard ratios (HRs) for brain tumor for patients with OSA were 1.78 (95% confidence interval [CI]: 1.42–2.21) in Model 1 (not adjusted with any covariate) and 1.67 (95% CI: 1.34–2.09) in Model 2 (adjusted for income level, diabetes, hypertension, dyslipidemia, and COPD). In subgroup analysis by gender, the odds ratios (OR) of OSA were 1.82 (95% CI: 1.41–2.33) in men and 1.26 (95% CI: 0.74–2.03) in women. The ORs were 1.97 (95% CI: 1.15–3.24) in the older (age ≥ 65 years) group, 1.66 (95% CI: 1.25–2.17) in the middle-aged (40 ≤ age < 65 years) group, and 1.41 (0.78–2.44) in the young (20 ≤ age < 40 years) group. In conclusion, OSA may increase the incidence of brain tumors.

## Introduction

Brain tumor is defined as a malignant neoplasm that develops in the tissues of the brain [1]. The various symptoms of brain tumor include headaches, focal neurological deficits, personality changes, partial or generalized seizures, confusion, and altered level of consciousness [2]. Although primary brain tumor is relatively rare, malignant neoplasm of the brain is a serious etiological factor for cancer morbidity and mortality [1–3]. According to the worldwide cancer incidence and mortality report, malignant tumors of the brain and central nervous system accounted for 1.8% of new cancer diagnoses (256,000 new cases) and 2.3% of cancer deaths (189,000 deaths) in 2012 [4]. Based on Korean cancer statistics in 2014, the age-standardized cancer incidence and mortality rates (per 100,000) of brain and central nervous system tumors were 2.7% and 1.7%, respectively [5].

Obstructive sleep apnea (OSA) is defined as a sleep disorder in which signs and symptoms (e.g., nonrestorative sleep, sleepiness, fatigue, breath-holding, and frequent snoring) occur in conjunction with at least five respiratory disorders (e.g., apnea, hypopnea, and respiratory effort-related arousal) per hour of sleep based on the sleep test [6, 7]. Although the exact mechanism of upper airway collapse remains unknown, it is possible that anatomical factors (e.g., craniofacial structural anomalies, and soft tissue enlargements), neuromuscular factors (e.g., ventilatory-control abnormalities, and decreased muscle tension), obesity, and factors related to the aging process are involved [8, 9]. Untreated OSA is associated with several deleterious health effects, including cardiovascular diseases (e.g., arrhythmias, ischemic heart disease, myocardial infarction, hypertension, congestive heart failure, stroke, and pulmonary hypertension), metabolic disorders (e.g., obesity, insulin resistance, dyslipidemia, and metabolic syndrome), and all-cause mortality [10–12].

Few clinical studies have investigated the potential impact of OSA on cancer mortality [13–15]. In addition, several studies reported the relationship between OSA and the incidence of malignant tumors [15–21]. However, a clear association between OSA and each malignant tumor has yet to be established. Therefore, the purpose of this study was to ascertain the effect of OSA on the incidence of malignant brain tumors using the Korea National Health Insurance Service (KNHIS) database.

## Materials and methods

### Data source

All Koreans are covered by the KNHIS [22]. The KNHIS reviews both inpatient and outpatient claims, including demographic data, diagnoses, direct medical costs, prescription records, and procedures. Each individual has a unique Korean resident registration number, which eliminates the possible risk of duplication or omission when evaluating the data. The KNHIS dataset manages claims based on the Korean Standard Classification of Diseases, sixth edition (KCD-6), a modified version of the International Classification of Diseases, 10th edition (ICD-10). Any investigator can use the KNHIS data if the clinical investigation protocols are approved by the official review committee.

### Study population and design

**Fig 1:**
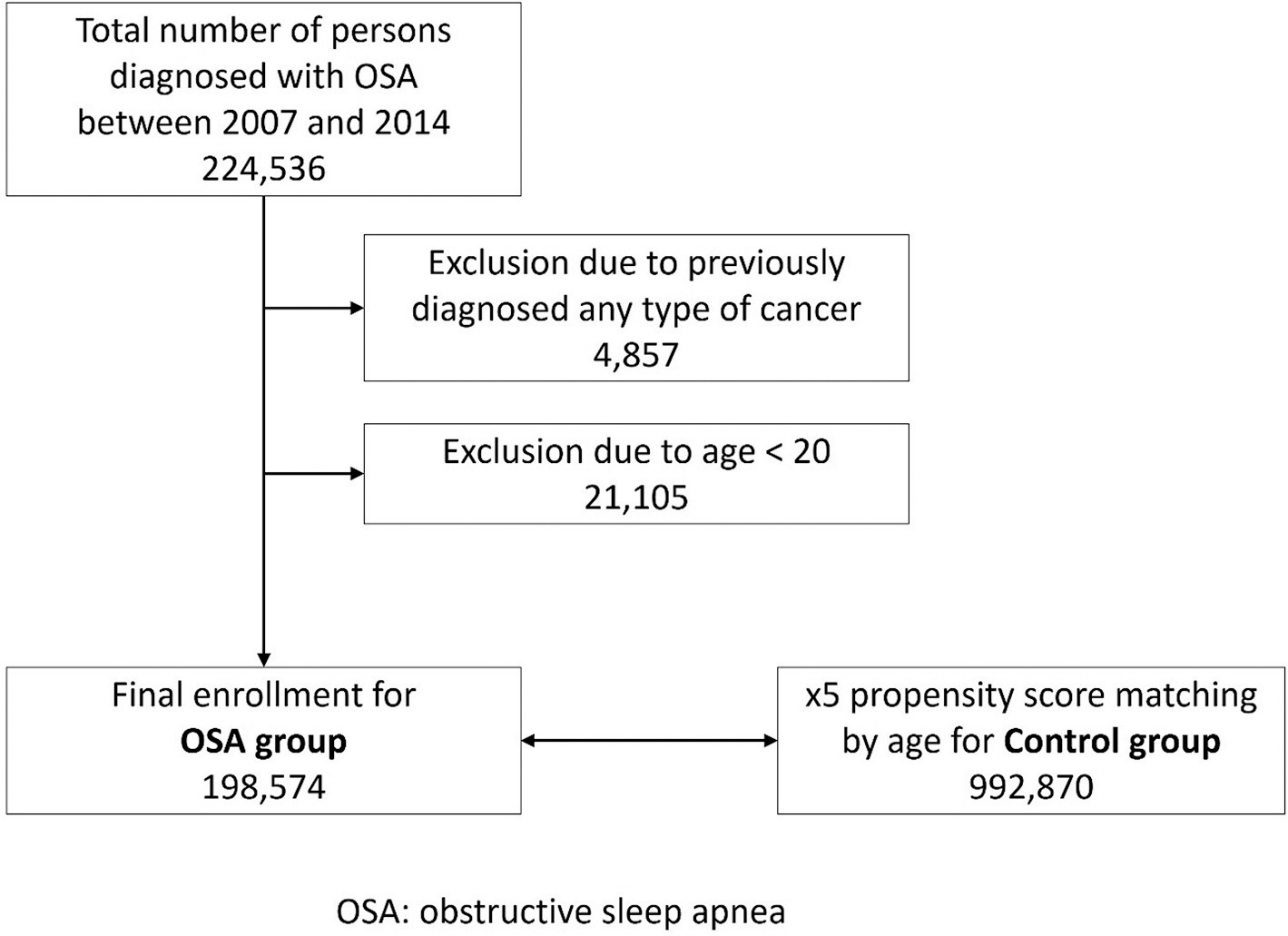
Flow chart of patient enrollment.

The study included all adult patients aged ≥ 20 years with newly diagnosed OSA (G47.30) between 2007 and 2014. Propensity score matching based on gender and age of individuals not diagnosed with OSA was used to select the controls [23]. The total number of individuals in the control group was five times that of the patients in the OSA group. The incidence of newly diagnosed malignant brain tumor was the primary endpoint. This study tracked subjects until December 31, 2015 based on ‘person-year at risk’ until brain tumors developed or patients were right-censored at the end of the follow-up period. They were also censored if they died. Since the entire population is insured nationally, the follow-up loss is not evaluated realistically. Patients diagnosed with any type of malignant tumor before enrollment were excluded. Fig 1 presents a flow chart of the study enrollment.

### Data collection

The following baseline data were gathered from the KNHIS dataset: age (years), gender, and income level (the lowest quintile). We also collected information related to comorbidities (e.g., diabetes, hypertension, dyslipidemia, stroke, chronic obstructive pulmonary disease, and ischemic heart disease) based on insurance claims data. The working definitions of diseases based on the insurance claims data are presented in Table 1.

**Table 1:** Working definitions based on insurance claims data.

Disease	Working definition
Obstructive sleep apnea	At least one claim under ICD-10 code G47.3
Brain tumor	At least one claim under ICD-10 code C71 and registered as a cancer patient in the National Medical Expenses Support Program.
Diabetes	At least one claim per year for the prescription of anti-diabetic medication under ICD-10 code E11-14.
Hypertension	At least one claim per year for the prescription of anti-hypertensive medication under ICD-10 code I10-13 or I15.
Dyslipidemia	At least one claim per year for the prescription of anti-dyslipidemic medication under ICD-10 code E78.
Stroke	At least one claim under ICD-10 code I63 or I64.
COPD	At least one claim under ICD-10 code J41, J42, J43, or J44.
IHD	At least one claim under ICD-10 code I20, I21, I22, I23, I24, or I25.

ICD, International Classification of Diseases; COPD, chronic obstructive pulmonary disease; IHD, ischemic heart disease.

### Statistical analysis

Data are displayed as the mean ± standard deviation for age and as proportions for the remaining categorical variables. Student’s *t*-test (continuous variables) or the χ^2^ test (categorical variables) was used to compare the two groups. The cumulative incidence was plotted graphically to easily compare the incidence of brain tumors in the OSA and the control groups. The Cox proportional-hazards model was utilized to calculate the hazard ratios (HRs) of brain tumor for patients with OSA. We applied two different models: Model 1 (not adjusted by any covariate) and Model 2 (adjusted for income level, diabetes, hypertension, dyslipidemia, and COPD). Furthermore, the univariate odds ratio (OR) was estimated based on gender and age. The outcomes are presented as HR (or OR) and 95% confidence interval (CI). We performed all statistical analyses using SAS version 9.4 (SAS Institute, Cary, NC, USA) and R version 3.2.3 (The R Foundation for Statistical Computing, Vienna, Austria).

### Ethical approval

All clinical investigation protocols were reviewed and approved by the Institutional Review Board of Soonchunhyang University Bucheon Hospital (SCHBC 2020-09-012). The current study was exempt from the requirement for informed consent since data available in the public domain was used. All methods were performed according to relevant regulations and guidelines.

## Results

A flow chart of patient enrollment is displayed in Fig 1. A total of 49,570,064 individuals were enrolled in the KNHIS in 2007. The current study data for the first year are available, and the numbers are similar to those of each subsequent year until 2014. There were 198,574 patients who were newly diagnosed with OSA between 2007 and 2014. A total of 992,870 individuals were selected as the control group. The average follow-up duration was 4.8 ± 2.3 years.

### Comparison between the OSA and control groups

Demographics of patients with OSA and controls are presented in Table 2. The age and gender of the control subjects were matched with those of the patients with OSA, whereas the other parameters showed relative differences. The income level of the patients with OSA was slightly higher, and all the other comorbidities (e.g., diabetes, hypertension, dyslipidemia, stroke, chronic obstructive pulmonary disease, and ischemic heart disease) were relatively common in patients with OSA.

**Table 2:** Demographic characteristics of OSA patients and controls.

	OSA	Controls	*P-*value
Total number	198,574 (100.0)	992,870 (100.0)	
Follow-up duration (years)	4.5 ± 2.3	4.5 ± 2.3	1.000
Mean age (years)	45.0 ± 13.3	45.0 ± 13.3	1.000
Age ≥ 65 years	15,123 (7.6)	75,615 (7.6)	1.000
Men	152,801 (77.0)	764,005 (77.0)	1.000
Income in the lowest quintile	34,005 (17.1)	222,002 (22.4)	<0.001
Diabetes	14,375 (7.2)	58,697 (5.9)	<0.001
Hypertension	47,746 (24.0)	144,766 (14.6)	<0.001
Dyslipidemia	33,398 (16.8)	86,233 (8.7)	<0.001
Stroke	9,221 (4.6)	22,000 (2.2)	<0.001
COPD	31,075 (15.6)	94,538 (9.5)	<0.001
IHD	34,851 (1.8)	8,478 (0.9)	<0.001

OSA, obstructive sleep apnea; COPD, chronic obstructive pulmonary disease; IHD, ischemic heart disease.

### The cumulative incidence of brain tumor

**Fig 2:**
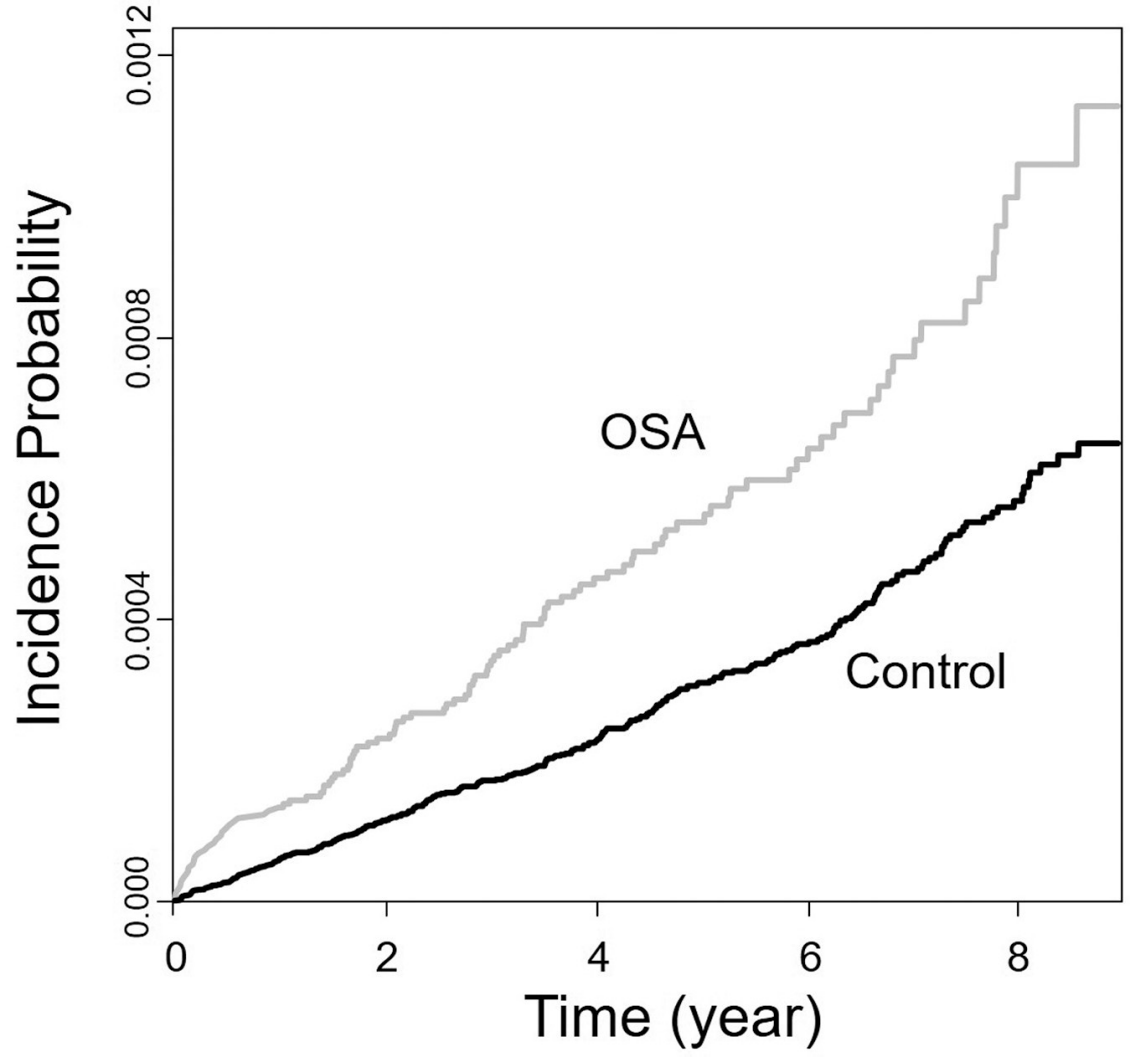
Cumulative incidence of brain tumor. The incidence of brain tumor was higher in the OSA group than in the control group.

The cumulative incidence of brain tumors among the OSA and control groups is plotted graphically as shown in Fig 2. Brain tumors occurred more frequently in the OSA groups compared to the control group.

### HR for brain tumor in the OSA group

The HR for brain tumors in the OSA group is presented in Table 3. The Cox proportional-hazards model revealed that the HR for brain tumors in the OSA group was significantly high in both models. The HRs were 1.78 (95% CI: 1.42–2.21) in Model 1 (not adjusted by any covariate) and 1.68 (95% CI: 1.34–2.09) in Model 2 (adjusted for income level, diabetes, hypertension, and dyslipidemia). The OR for brain tumors by gender is presented in Table 4. The OR for men (but not women) was significantly high. The OR for brain tumor by age group is presented in Table 5. The OR tended to increase with age, but was not statistically significant.

**Table 3:** Hazard ratio for brain tumor for patients with OSA.

	Number	Event	Rate	Model 1^a^	Model 2^b^
Controls	992,870	304	0.064	1	1
OSA	198,574	108	0.114	1.78 (1.42–2.21)	1.67 (1.34–2.09)

() means 95% confidence interval / OSA, obstructive sleep apnea.

^a^Model 1: not adjusted.

^b^Model 2: adjusted by income level and diabetes, hypertension, dyslipidemia, and COPD.

**Table 4:** Univariate odds ratios for brain tumor by gender.

Age (years)	Men	Women
Controls	1	1
OSA	1.82 (1.41–2.33)	1.26 (0.74–2.03)

() means 95% confidence interval / OSA, obstructive sleep apnea.

**Table 5:** Univariate odds ratios for brain tumor by age group.

Age (years)	20 ≤ Age < 40	40 ≤ Age < 65	65 ≤ Age
Controls	1	1	1
OSA	1.41 (0.78–2.44)	1.66 (1.25–2.17)	1.97 (1.15–3.24)

() means 95% confidence interval / OSA, obstructive sleep apnea.

## Discussion

In summary, 1) brain tumors occurred more frequently in patients diagnosed with OSA than in the control group, and 2) this trend was more pronounced in men and persisted with age. To the best of our knowledge, this is the second cohort study providing important evidence supporting a significant relationship between OSA and the incidence of brain tumors [24].

In this study using the KNHIS database, the HR of OSA on brain tumor was 1.78 (95% CI: 1.42–2.2) in Model 1 (not adjusted by any covariate). The HR was 1.67 (95% CI: 1.34–2.09) after adjusting for income level, diabetes, hypertension, dyslipidemia, and COPD (Model 2). These outcomes are consistent with those of a previous study that suggested a potential causal link between OSA and malignant brain tumors [24]. Chen et al. analyzed the risk factors for brain tumor in patients with OSA based on the claims dataset of Taiwan’s National Health Institute program and found that the cumulative hazard of brain tumors was significantly higher in patients with OSA than in control subjects (1.71 [95% CI: 1.06–2.75]) [24].

There are several potential pathogenetic mechanisms associated with the development of brain tumors in OSA, including sleep fragmentation, chronic systemic inflammation, immune dysfunction, intermittent hypoxia, and oxidative stress [25]. It is well known that tumor cells under hypoxic conditions lead to cellular processes resulting in the survival of these cells, as well as short- and long-term adaptation, such as angiogenesis, metastasis, and non-response to radiotherapy or chemotherapy [26]. In particular, intermittent hypoxia induces the activation of diverse transcription factors, including nuclear factor (NF) of activated T cells, NF-κB, activator protein-1, hypoxia-inducible factor-1, and NF (erythroid-derived 2)-like 2, and the expression of specific genes associated with long-term adaptation [27, 28]. In addition, intermittent hypoxia results in increased oxidative stress, chronic inflammation, and DNA damage by producing reactive oxygen species and promotes oncogenesis and migration of malignant tumor cells [27]. The dysregulation of the immune system caused by OSA may also be associated with an increase in malignant tumor incidence [29]. Gaoatswe et al. investigated the effect of OSA on the frequency of invariant natural killer T (iNKT) cells that play a critical role in tumor immunity and showed that the frequency of circulating iNKT cells was decreased in patients with severe OSA [30]. Moreover, the numbers of circulating iNKT cells correlated inversely with apnea-hypopnea index and the severity of hypoxemia during sleep estimated by oxygen desaturation index and percentage of sleep time with SpO_2_ < 90% [30].

In subgroup analysis, the HR for brain tumors was 1.82 (95% CI: 1.41–2.33) in men and 1.26 (95% CI: 0.74–2.03) in women. The differences between men and women involve OSA-related upper airway anatomy, prevalence, clinical manifestations, consequences, and treatments [31]. In addition, similar to OSA, brain tumors may differ in gene expression, immune function, growth, metabolism, and homeostatic response to stressors based on gender [32]. Since a variety of factors may be implicated, the impact of gender differences on the relationship between OSA and the development of brain tumor is still unclear. Further studies are needed to elucidate the mechanisms and pathways involved.

The HR tended to vary with age. The ORs for brain tumor were the highest in the older (age ≥ 65 years) patients (1.97 [95% CI: 1.15–3.24]), followed by middle-aged (40 ≤ age < 65 years) patients (1.66 [95% CI: 1.25–2.17]), and young (20 ≤ age < 40 years) patients (1.41 [95% CI: 0.78–2.44]). The results of this study are in line with those of previous studies. Sillah et al. [20] evaluated the potential relationship between OSA and cancer incidence by estimating age–sex standardized cancer incidence ratios (SIRs) using data derived from a cohort of subjects with OSA diagnosis in a population-based malignant tumor registry. The overall cancer incidence was increased in patients with OSA (SIR 1.26 [95% CI: 1.20–1.32]) and the cancer incidence was the highest in older patients (age ≥ 60 years) with OSA (SIR 2.43 [95% CI: 2.26, 2.60]) compared to the general population [20].

The current study was undertaken to compare brain tumor incidence between OSA and a control group using the claims data of the KNHIS dataset, which allowed access to an increased number of subjects. However, this study has several limitations. First, there are several possible confounding factors involving both OSA and malignant brain tumors, such as hereditary background, cigarette smoking status, alcohol intake, and obesity. However, data pertaining to these confounding factors could not be obtained because this study was based on claims data. In addition, the analysis based on the type or stage of brain tumor was not performed. Second, this study does not report the accuracy of OSA diagnosis and the severity of apnea-hypopnea index. Third, the study results do not represent all patients since we used only a Korean population-based dataset.

## Conclusion

The incidence of brain tumor may be increased in patients with OSA compared to the controls. The results reveal a significant relationship between OSA and brain tumors.
